# Oral Ezatiostat HCl (TLK199) and Myelodysplastic syndrome: A case report of sustained hematologic response following an abbreviated exposure

**DOI:** 10.1186/1756-8722-3-16

**Published:** 2010-04-23

**Authors:** Fahd Quddus, Jessica Clima, Helen Seedham, Ghulam Sajjad, Naomi Galili, Azra Raza

**Affiliations:** 1 Dept of Hematology and Oncology, University of Nebraska Medical Center, Omaha, NE: 68198 USA; 2 St. Vincent's Comprehensive Cancer Center, 325 West 15th Street, New York, New York 10011 USA

## Abstract

Treatment options for patients with lower risk non-del(5q) myelodysplastic syndromes (MDS) who fail erythroid stimulating agents are restricted to one of the hypomethylating drugs with an expected response rate of ~50%. Ezatiostat HCl, an agent with the potential for producing multi-lineage responses in this population is currently in clinical investigation phase. This case report describes a 77 year old male who received less than two cycles of therapy with ezatiostat HCl which had to be aborted due to intolerable side effects, but which produced a sustained normalization of all three blood counts. This trilineage response has now lasted for more than a year. Interestingly, the patient began with a del(5q) abnormality and responded briefly to lenalidomide. Upon relapse of the anemia, a bone marrow showed the disappearance of the del(5q) but the appearance of a new clonal abnormality t(2;3). Given that the patient had a complete cytogenetic response to a truncated exposure to lenalidomide followed by a trilineage response to an even briefer course of ezatiostat HCl suggests a potential role for ezatiostat HCl in del(5q) patients who relapse following lenalidomide.

## Background

Myelodysplastic syndromes (MDS) are a group of bone marrow stem cell disorders which generally affect the elderly (median age of 70 years) and present with the paradox of a variable cytopenia with a cellular marrow. Prognostically, these syndromes are broadly divided into those having a lower or higher risk of transformation to acute myeloid leukemia (AML) on the basis of three variables; the number of cytopenias, percentage of bone marrow blasts and cytogenetics [1]. Consequently, the course of MDS and response to therapy vary between the prognostic categories. Allogeneic stem cell transplant offers the only potential for cure to these patients; unfortunately, because of the advanced age, this is an option for only a select few. The primary goal of palliative therapy in lower risk patients is to improve the cytopenias while that for the higher risk MDS, in addition, is to arrest the expanding population of blasts [2]. Currently, there are three FDA approved drugs; lenalidomide is indicated for the treatment of transfusion dependent MDS patients with del(5q) and lower risk disease while the two hypomethylating agents (azacytidine and decitabine) are approved for all categories. With the exception of del(5q) patients, the response rate is approximately 50%, highlighting the need for clinical trials of new agents.

Ezatiostat (HCl) is a glutathione analog which has demonstrated in vivo hematopoietic stimulatory activity in several clinical trials is one such novel agent currently undergoing clinical investigation [3-7]. Here, we report the case of a patient with a lower risk MDS who responded unexpectedly well to an abbreviated course of this investigational agent.

## Case Presentation

A 77-year-old Caucasian male with a history of reflux esophagitis requiring fundoplication was found to be pan-cytopenic in December 2007 (WBC 3.0 × 10^3^/μL, hemoglobin 9.5 g/dL, and platelet count 115,000/μL). A bone marrow (BM) biopsy on 3/17/08 was consistent with a diagnosis of MDS with a mildly hypocellular marrow, minimal dyserythropoiesis, less than 5% blasts and cytogenetics showing 17 of 20 metaphases with a del(5q)(q13q33). He was subsequently started on lenalidomide and responded initially with the hemoglobin level increasing to 11-12 gram range and platelet counts increasing to 130,000 - 150,000 range. The counts decreased within 6 months of an initial response and a repeat BM in 08/27/08 revealed a normocellular marrow with dysplastic changes in the myeloid and erythroid series and mildly increased blasts at 6%. Interestingly, the del(5q) abnormality had disappeared, however, a new t(2; 3) translocation in 16 of 20 metaphases was seen. The patient discontinued lenalidomide, and offered therapy with hypomethylating agents which he refused. The patient was subsequently referred to us in September 2008. He had an erythropoietin level of 1840, serum iron 44, ferritin 112, TIBC 222, folic acid > 24, and vitamin B12 level of 672. His peripheral blood was negative for the JAK2 V617F point mutation, and showed normal expression of CD55 and CD59. The patient agreed to participate in a randomized, open label, multi-center phase 2 study comparing two dose schedules of Ezatiostat HCl for Low to Intermediate -1 risk MDS and was randomized to receive ezatiostat at 1500 mg p.o. b.i.d. for 14 days of a 21-day cycle for a maximum of 24 weeks. The patient started his first cycle on 10/23/08, with his second cycle beginning on 11/13/08. However, five days into his second treatment cycle he was withdrawn from the study (11/18/08) due to a grade 3 acute gastritis and biopsy proven candida esophagitis. He was started on proton pump inhibitors and oral antifungal therapy to which he responded well. The gastritis resolved completely with medical management within two weeks.

**Figure 1:**
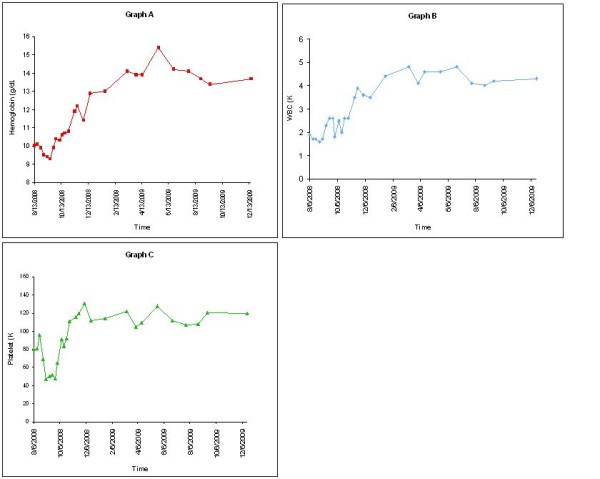
**Graph A: Hemoglobin curve**. Graph B: White blood cell curve. Graph C: Platelet curve.

Figure 1 shows the striking improvement in all three blood counts beginning with ezatiostat HCl treatment initiation and continuing to remain high a year post therapy. These results are impressive when one considers that the patient received treatment for less than one and half cycles. Pre-study CBC showed a baseline WBC of 1.5 to 2.5 × 10^3^/μL, hemoglobin of 9 to10 g/dL, and platelet count of 50,000 to 100,000/μL. Post study, patient's CBC has remained steady with a WBC of approximately 4 to 4.5 × 10^3^/μL, hemoglobin above 13 grams/dL, and a platelet count of 100,000 to 130,000/μL. He has not required any further therapy for his underlying MDS including any use of growth factors. The patient declined a repeat bone marrow exam.

Ezatiostat HCl, a glutathione analog prodrug, has shown significant stimulatory and prodifferentiating activity in vitro in human bone marrow progenitor cultures, as well as in several in vivo preclinical models of myelopoiesis [3-5]. Initially ezatiostat hydrochloride liposomes for injection (IV formulation) were evaluated in MDS in a Phase 1-2a study with observed good tolerability and HI responses [6]. An oral formulation of ezatiostat HCl tablets in a phase 1 study was recently published [7]. In this study no dose-limiting toxicities were observed. The most common grade 1 or 2, respectively, treatment-related adverse events were non-hematologic: nausea (56%, 9%), diarrhea (36%, 7%), vomiting (24%, 7%), abdominal pain (9%, 0%), constipation (4%, 9%), anorexia (3%, 7%), and dyspepsia (3%, 7%). Forty five patients were treated on the Phase I dose escalation study and seventeen hematologic improvement (HI) responses were observed. While HI was seen at even the lowest dose, 11/17 HI responses were at doses between 4000 to 6000 mg/day. HI responses included 3 bilineage and 1 complete cytogenetic response with improved transfusion requirements and in some cases transfusion independence. Currently a phase 2 study to determine the optimal dosing schedule for oral ezatiostat HCl is underway, and our patient was on one arm of this study.

We report this unique case, as the patient received therapy with oral ezatiostat HCl tablets for less than 2 cycles, but has shown an impressive hematologic response that has lasted for over a year. Interestingly, this patient presented with a del(5q) abnormality which disappeared after a short course of lenalidomide, only to be replaced by a new clonal abnormality t(2;3) upon relapse. Despite resistance to lenalidomide and the appearance of this new clone, the patient experienced a durable trilineage response to a very short and aborted course of ezatiostat HCl suggesting either an exceptionally responsive disease or a potential role for ezatiostat HCl in the treatment of patients who relapse following lenalidomide therapy.

## Conclusion

In conclusion, this case highlights two important observations; first that even a brief exposure to ezatiostat HCl produced a striking and sustained hematologic response, and secondly that this occurred in a patient who relapsed after lenalidomide therapy. As MDS is a heterogeneous disease, it would be important to identify the subset of MDS patients, who like our patient above, may receive clinical benefit from this agent.

## Consent Statement

Written informed consent was obtained from the patient when he was accrued on the ezatiostat HCl trial. A copy of this informed consent which includes permission for publication, has been submitted to the Editors. Any information that could identify our subject has been withheld.

## Competing interests

The authors declare that they have no competing interests.

## Authors' contributions

FQ reviewed the charts and contributed to writing the manuscript. JC and HS were responsible for patient care. GS and NG were responsible for data collection, review and manuscript preparation. AR is the Principal Investigator of the trial and was involved in all aspects of patient care, data analysis and manuscript preparation. All authors read and approved the final manuscript.
